# Laser-induced growth of nanocrystals embedded in porous materials

**DOI:** 10.1186/1556-276X-8-266

**Published:** 2013-06-06

**Authors:** Bruno Capoen, Abdallah Chahadih, Hicham El Hamzaoui, Odile Cristini, Mohamed Bouazaoui

**Affiliations:** 1 Laboratoire de Physique des Lasers, Atomes et Molécules (CNRS, UMR 8523), IRCICA (USR CNRS 3380), CERLA (FR CNRS 2416), Bâtiment P5, Université Lille 1-Sciences et Technologies, Villeneuve d’Ascq Cedex, 59655, France

**Keywords:** Mesoporous silica, Laser irradiation, Metal, Semiconductor, Nanocrystals

## Abstract

Space localization of the linear and nonlinear optical properties in a transparent medium at the submicron scale is still a challenge to yield the future generation of photonic devices. Laser irradiation techniques have always been thought to structure the matter at the nanometer scale, but combining them with doping methods made it possible to generate local growth of several types of nanocrystals in different kinds of silicate matrices. This paper summarizes the most recent works developed in our group, where the investigated nanoparticles are either made of metal (gold) or chalcogenide semiconductors (CdS, PbS), grown in precursor-impregnated porous xerogels under different laser irradiations. This review is associated to new results on silver nanocrystals in the same kind of matrices. It is shown that, depending on the employed laser, the particles can be formed near the sample surface or deep inside the silica matrix. Photothermal and/or photochemical mechanisms may be invoked to explain the nanoparticle growth, depending on the laser, precursor, and matrix. One striking result is that metal salt reduction, necessary to the production of the corresponding nanoparticles, can efficiently occur due to the thermal wrenching of electrons from the matrix itself or due to multiphoton absorption of the laser light by a reducer additive in femtosecond regime. Very localized semiconductor quantum dots could also be generated using ultrashort pulses, but while PbS nanoparticles grow faster than CdS particles due to one-photon absorption, this better efficiency is counterbalanced by a sensitivity to oxidation. In most cases where the reaction efficiency is high, particles larger than the pores have been obtained, showing that a fast diffusion of the species through the interconnected porosity can modify the matrix itself. Based on our experience in these techniques, we compare several examples of laser-induced nanocrystal growth in porous silica xerogels, which allows extracting the best experimental conditions to obtain an efficient particle production and to avoid stability or oxidation problems.

## Review

### Introduction and background

Linear and nonlinear optical properties of metal [1,2] and semiconductor [3,4] nanoparticles are now well-known, and numerous applications [5,6] have been envisaged for ages. In the past decade, we have witnessed huge advances in chemical synthesis of nanoparticles (NPs), especially metal NPs that have found applications in plasmonics-based biosensors and surface-enhanced Raman spectroscopy (SERS) detectors [7,8] and biological fluorescent labeling [9]. Up to now, the commercial use of NPs, still limited to colloidal solutions or thin films, is always based on the linear optical properties of metal clusters (the so-called surface plasmon resonance (SPR)) or of semiconducting nanocrystals (tunable exciton light emission). In order to exploit the now demonstrated nonlinear optical properties [10,11] of such quantum dots and to go further towards photonics applications (lasers, optical fibers), we now need to embed the nanocrystal in vitreous matrices, if possible, in a localized manner. However, in the state of the art, when nanoparticles can be produced in glasses or other transparent matrices, it is essentially without space selectivity. Through photosensitivity effects, the laser techniques have been demonstrated for many years to be efficient in structuring the matter and more particularly in Bragg embodiment in optical waveguides [12]. Either isotropic or anisotropic linear refractive index changes (up to a few 10^−3^) have been obtained under laser irradiation, due to densification processes or stoichiometric defects in hydrogen-loaded germanosilicate glasses. Furthermore, where pulsed lasers are used with higher fluence or high peak power density, larger densification and even damaging can occur, yielding a large refractive index contrast, a seducing application of which could be imagined in the topical domain of data storage [13]. Finally, at the highest power density, the intense electric field may blast the matter, producing surface corrugation or microbubbles. With regard to the production of NPs using a laser, apart from the now well-known pulsed-laser deposition and laser pyrolysis techniques, a recent method based on laser-induced transfer of molten metal allowed to deposit one unique small gold particle (20 nm diameter) on a surface [14]. All of these techniques are however inappropriate for doping a bulk sample with NPs.

Our purpose is to show that a suitable combination of doping and laser techniques makes it possible to obtain localized NP growth in vitreous matrices. The theoretical space resolution of a pattern of NP, photoinscribed using a simple microscope objective, is roughly limited in the Abbe theory by:

(1) $$d_{\lim}=\frac{\lambda }{2\mathrm{NA}}$$

where *λ* is the radiation wavelength, and *NA* is the microscope numerical aperture.

Moreover, considering the inevitable atomic diffusion in the glass under high laser power densities, this resolution is finally comparable with that of a phase mask technique (approximately 0.5 μm). Hence, it would be an illusion to believe in achieving the creation of one unique particle (the grail of nanoscience), but at least the wavelength scale can be reached, and more importantly, the number of possible designs is virtually infinite at the micron scale.

**Figure 1:**
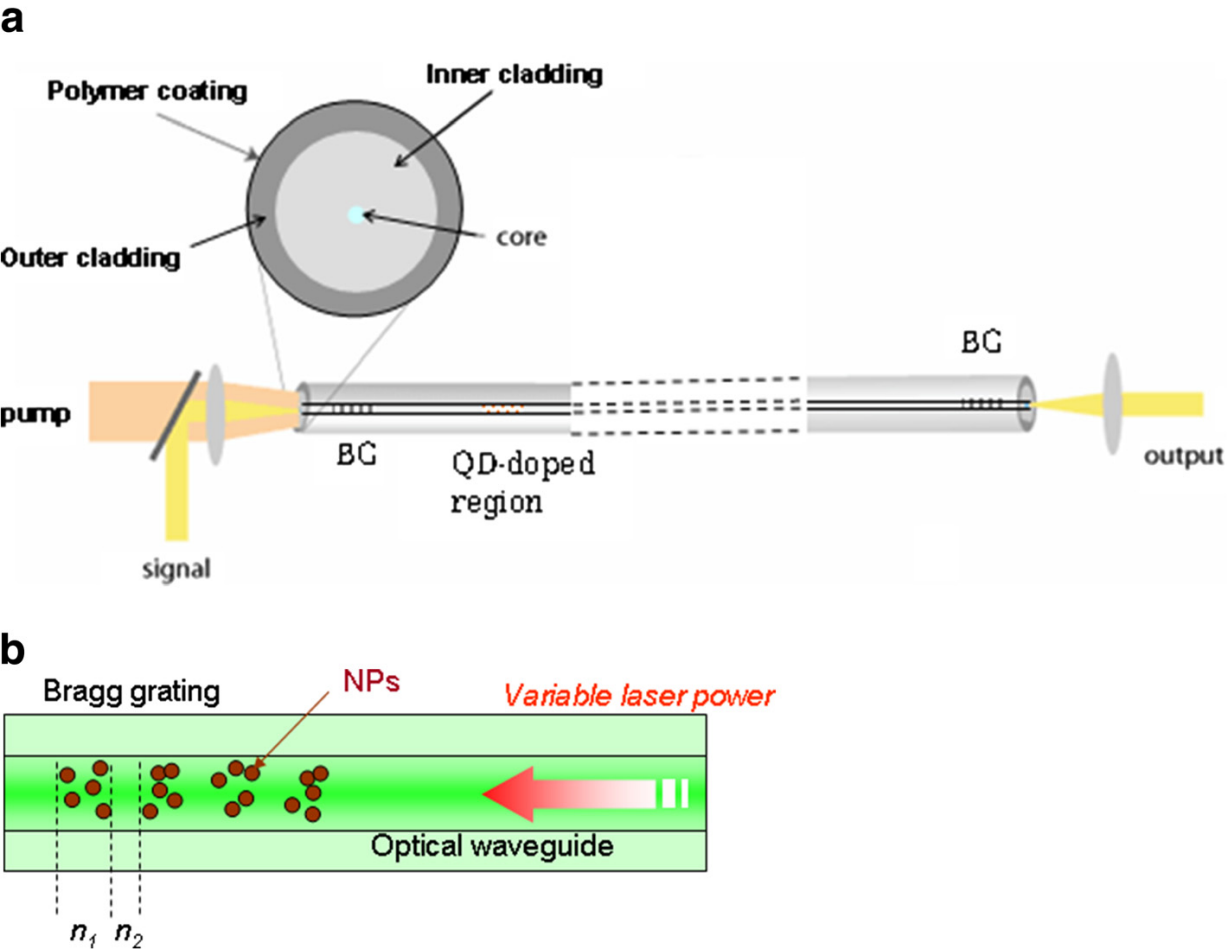
**Examples of new-generation optical device concepts using NP in a fiber core.** (**a**) Quantum dot-based laser consisting in a NP-doped core region inside an optical cavity using Bragg gratings (BG). The pump light at any wavelength lower than the exciton wavelength can be guided in the inner cladding, interacting with the QD by leaking modes. (**b**) All-optical control of the properties of a Bragg grating containing periodic arrangement of NP.

While a first fundamental objective is to understand the physico-chemical processes involved in the NP growth under laser irradiation, an obvious target is also represented by applications like configurable photonic devices and especially by doped fibers. Indeed, under the assumption that doping dense glasses with the NP precursors and controlling the NP growth under laser irradiation are possible, one can imagine such an experiment where NPs are created in the pre-doped fiber core after its drawing, by exposing it to the laser beam. The local precipitation of NPs in a fiber core may itself be useful in laser technology, where NPs can act for example as emitters (Figure 1a) or saturable absorbers. Another example of application idea was given in a patent deposited by Alcatel [15] in 2004. It consists in creating a Bragg grating by doping periodic zones with NPs (Figure 1b), then using the enhanced Kerr optical effect of the composite zones to optically control either the reflection wavelength or the filter contrast, two parameters depending on the effective refractive index. This prospect, as well as the one related to other applications like photochromic display systems [16], has substantially increased the interest in using laser irradiation to generate particles in a glass and also in a xerogel matrix. What is called a xerogel here can be presented as a porous glassy phase with interconnected pores [17]. Hence, atoms of NP precursors have a higher mobility than in a dense glass, facilitating the NP formation without any specific heat treatment, contrary to the case of dense glasses. Indeed, concerning metal nanoparticles, since the pioneer work of Qiu et al. [18] in 2002, many other studies have dealt with precipitating gold, silver, and even copper nanocrystals in dense melted glasses [19,20]. The principle is first to reduce metal cations by extracting electrons from the matrix using infrared femtosecond (fs) pulses. The high electric field of the pulses creates nonbridging oxygen holes and free electrons that can be trapped by metal ions [21]. A subsequent heat treatment is however necessary to give the metal atoms a sufficient mobility in the vitreous matrix, allowing their migration to the existing clusters [22] and yielding the formation of nanoparticles. In theory, the energy needed for this diffusion is much weaker in the case of a porous medium.

Alkoxide-derived inorganic xerogels have been recently shown as a much cheaper alternative to chemical vapor deposition methods for providing pure silica rods. Those porous silica rods could even be easily impregnated with doping solutions, then densified and drawn into microstructured fiber preforms after association with other silica rods [23]. Here, we review the different experimental configurations employed in our group (in Lille) to obtain space-localized metal or semiconductor NP in a bulk xerogel. The main objective is to help the reader compare and choose the best method, together with the adapted precursor for the space-selective growth of NPs. The criteria of this choice could be space resolution, high efficiency, particle size, stability, etc.

### Characteristics of materials and lasers

**Figure 2:**
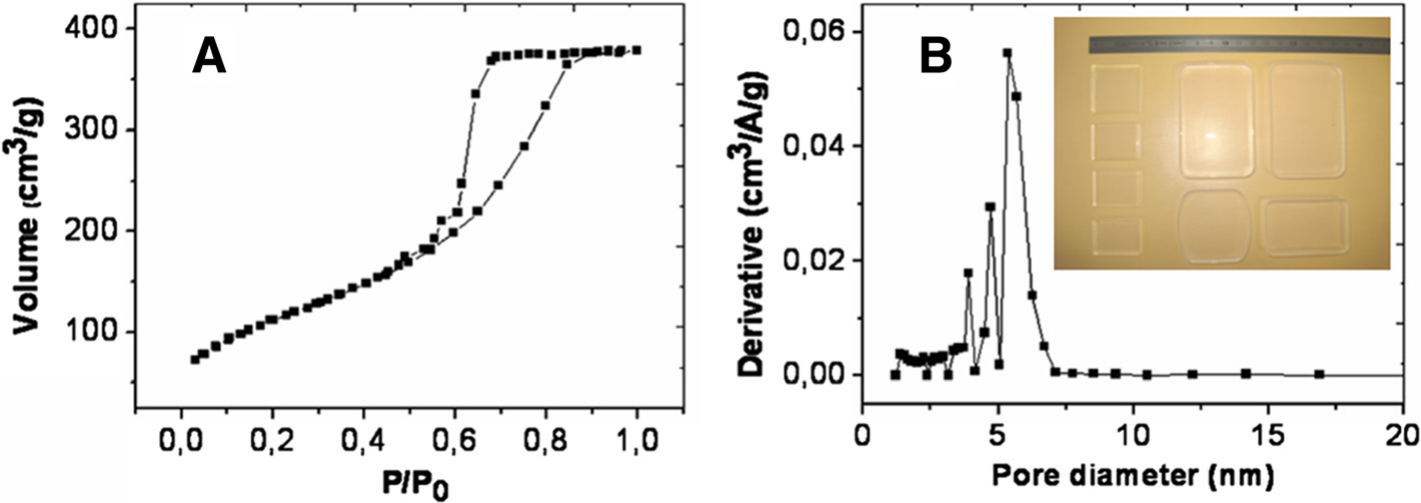
**Nitrogen adsorption-desorption isotherm of a typical base-catalyzed TMOS-derived xerogel (A) and the resulting pore size distribution (B).** As detailed in [15]. The inset shows the obtained transparent bulk samples.

Most of the raw samples mentioned throughout this work are pure bulk silica xerogels prepared using a tetramethyl orthosilicate (TMOS) precursor in a base-catalysis protocol [17]. Unless otherwise informed, these transparent xerogels present interconnected pores of average diameter of 5 to 6 nm, once stabilized at 850°C (Figure 2), which allows an efficient impregnation with a doping precursor solution. Metal doping precursors are generally salts (nitrate, acetate) dissolved in water or ethanol. Sulfur can be brought by an organosulfur compound (thiourea). The whole must be mixed in a homogeneous solution designed to seep into the xerogel porosity, which limits the precursor choice and concentration to the solubility threshold. The porous xerogels are immersed in the doping solution for 4 h, then taken out and dried at 50°C for several hours to remove solvents and to retain the precursor within the pores. The resulting doped xerogels are generally transparent or pale yellow.

**Figure 3:**
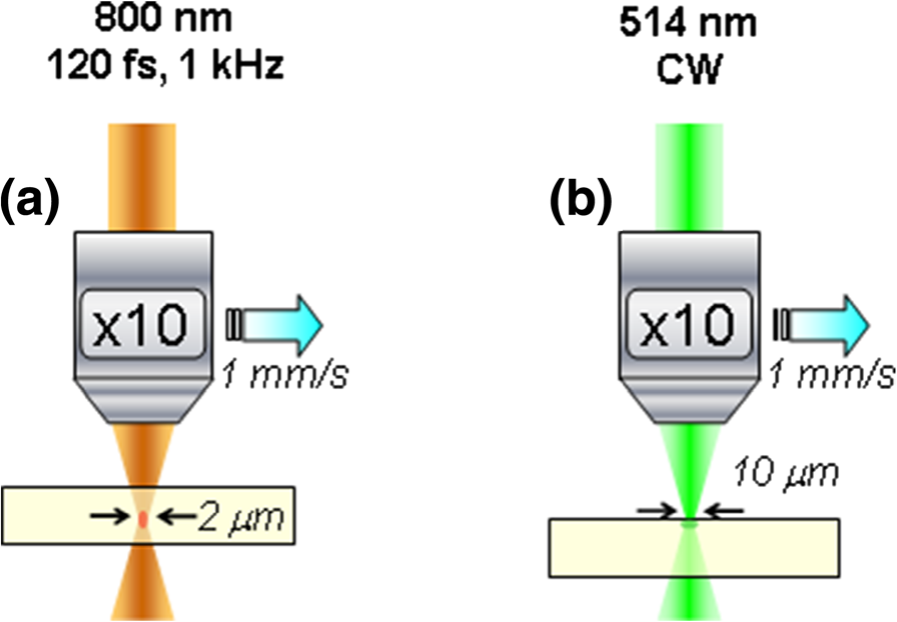
**Schematic drawings of the two main configurations used for the xerogel irradiation.** (**a**) With a femtosecond infrared laser and (**b**) with a CW visible laser. In both cases, the sample is mounted on a 3-axis stage allowing to draw motifs or dense arrays with a micrometer precision.

The employed lasers may be classified in two types according to their wavelengths and power densities. Exceptions aside, the doped xerogels present an optical absorption threshold between 300 and 400 nm, which means that infrared radiation (800 nm) cannot be absorbed with one photon. However, being given the high power density of femtosecond pulses, multiphoton absorption phenomena occur, which makes it possible to obtain 3D-localized effects in the bulk volume of a sample (Figure 3a). On the contrary, where continuous wave (CW) visible laser (514.5 nm) or pulsed UV laser is used [24], light is absorbed over a few microns (Figure 3b), even in the case of weak absorption, because once a few small particles are created, they begin to absorb light at this wavelength. Hence, 2D micropatterns can be imprinted only at or just beneath the sample surface.

### Metal nanoparticles in a xerogel

The localization of metal nanocrystals inside a glass matrix may be desired for applications based on the electric field enhancement, like SERS detection, for their sensitivity to environment [25] or for their optical nonlinearities [26].

#### Silver nanoparticles

**Figure 4:**
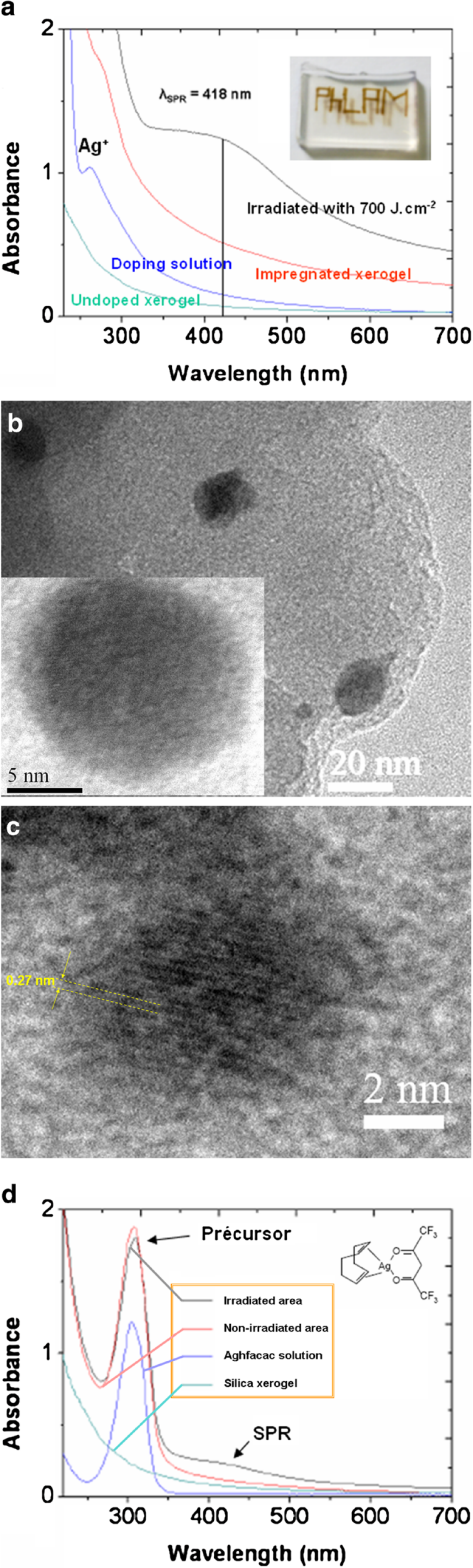
**Local growth of Ag-NP under CW laser irradiation at 514 nm.** (**a**) Optical absorption spectra of a sample doped with silver nitrate in various conditions and a photograph of the ‘written’ sample after irradiation. (**b**) Corresponding TEM images showing Ag-NP of large dimensions. A magnification of certain NP shows lattice planes attributed to metallic Ag. (**c**) Another HRTEM image showing atom interplanar distances corresponding to Ag_2_O. (**d**) Optical absorption spectra obtained with the precursor Aghfacac.

A first simple experiment consists in impregnating the porous silica xerogel with a low-concentrated aqueous solution of silver nitrate (AgNO_3_, 0.02 M) and then irradiating it with a CW argon laser at 514.5 nm. As summarized in Figure 3b, the sample is irradiated through a microscope objective, giving a spot of diameter of 10 μm, which is scanned on the sample at a speed of 1 mm/s to write or draw a motif or to cover a sufficient surface, in order to perform characterization experiments. As shown in Figure 4a, a brown color appears at the surface of the sample after depositing about 700 J/cm^2^. In the absorption spectra of the doping solution and of the doped xerogel before irradiation, the band at 260 nm can be attributed to Ag^+^ ions or to Ag_2_^+^ dimmer formation. In the spectrum of the irradiated zone, this band is replaced by a large band around 418 nm, ascribable to the SPR of silver NP (Ag-NP). The transmission electron microscopy (TEM) also reveals the presence of Ag-NPs in this zone (Figure 4b). The measured interplanar distance of about 0.2 nm corresponds well to the *d*_200_ distance of cubic silver structure. Particles do not really have a spherical shape, but more important is the NP diameter that can reach over 20 nm, namely a diameter larger than the mean pore size. Thus, it is obvious that a fast diffusion of Ag atoms occurs between the interconnected pores, and this fast process is prone to destroy or at least to rearrange the silica network in order to allow larger pores to be created. This result and the amplitude of the absorbance band are the signs of a rather efficient growth process, in connection with an efficient reduction process of the silver cations. Now, electrons involved in this reduction essentially come from the matrix. Actually, in a xerogel before its densification, the important specific surface area provides propitious conditions for the existence of a wide variety of defects, like oxygen vacancies or Si-OH dangling bonds [27,28]; these defects are sufficient to provide electrons under laser irradiation and to reduce the Ag^+^ ions liberated by the nitrate. However, this reduction process is not perfect because probable oxide phases (Ag_2_O) could also be detected by other TEM analysis (Figure 4c). This reflects the natural tendency of Ag-NP to be oxidized if they are not protected.

The silver precursor has a strong influence on the reduction process. To realize this, a more complicated molecule can be used, like silver hexafluoroacetylacetonate (1.5-cyclooctadiene), alias Aghfacac. Contrary to the silver nitrate, this precursor molecule is not entirely broken in the aqueous solution and presents several bonds between Ag and the organic groups. As a consequence, the energy density necessary to produce NP is multiplied by 2.5, and only a slight release of Ag^+^ ions occurs under the laser irradiation. This is the reason why the optical spectra exhibit a very weak SPR band after irradiation, contrary to the band at 307 nm ascribed to the precursor, which remains almost unchanged (Figure 4d). In other words, a nonnegligible amount of complementary thermal energy is necessary to obtain Ag^+^ ions from this precursor. This heat quantity, coming from the weak absorption of light by the matrix and by the precursor, is also used to grab electrons from the matrix defects.

#### Gold nanoparticles

**Figure 5:**
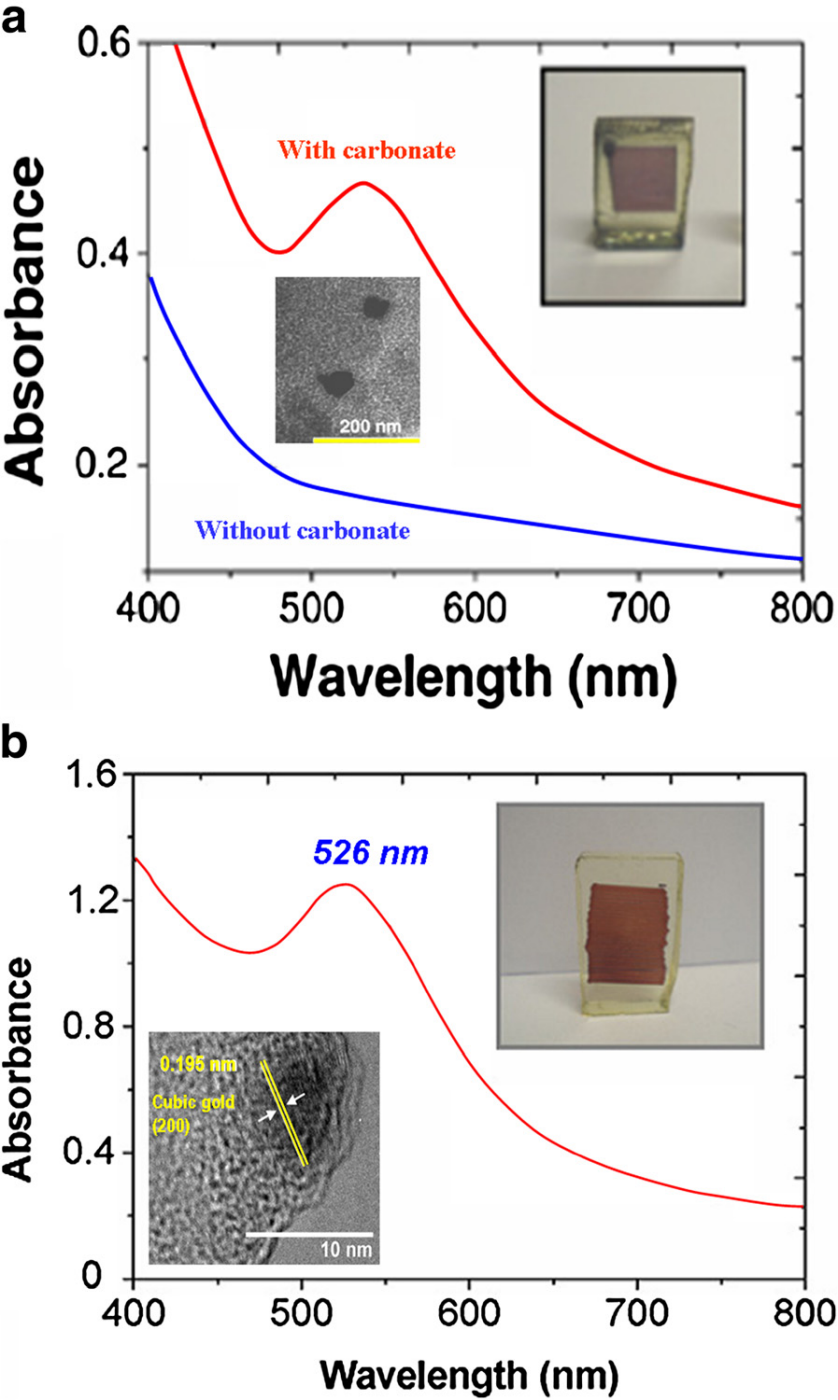
**Local growth of Au-NP in xerogels doped with HAuCl**_**4**_**.** (**a**) Optical absorption after fs irradiation. Photograph and TEM image obtained on a sample co-doped with sodium carbonate. (**b**) Optical absorption after CW irradiation. Photograph and TEM image obtained on a sample co-doped with sodium carbonate. (a and b) adapted from [29] and [30], respectively.

As already recalled, gold nanoparticles (Au-NP) had already been grown inside dense melted glasses with small amounts of gold oxide in the melt batch [18], achieving beautiful drawings under fs irradiation and after annealing at 550°C. The same can also be obtained in a porous silica xerogel by a 120-fs pulsed laser irradiation [29] with a cadency of 1 kHz and a mean power of 26 mW. The advantage of using such a porous matrix lies in the possibility of obtaining very localized doped patterns in only one step, that is to say without any further heat treatment. Tetrachloroauric acid (HAuCl_4_) may be used as a Au^3+^ precursor, but in this case, a sodium carbonate additive Na_2_CO_3_ is needed in the impregnation solution, as shown in Figure 5a where the SPR band of Au-NP is observed only in the sample with carbonate. The role of the additive has been explained to be a sensitizer role for the cation reduction [29]. In the present experimental conditions, the photoreduction process cannot be a pure thermal process, because if it was, a simple heat treatment would have given the same result on the same samples. Nevertheless, if a sample impregnated by a solution without carbonate is annealed at 120°C, Au-NP growth is clearly observed within a few minutes. Hence, the carbonate ion acts as an electron provider through a chemical reaction assisted by a multiphoton absorption implying at least three photons:

(2) $$3{\mathrm{CO}}_{3}{}^{2-}+\mathrm{nhv}\to 3/2{\;C}_{2}O_{6}{}^{2-}+3e^{–}+Q$$

where *nhv* designates the energy of *n* photons, and *Q* is the heat quantity given off by the reaction. The huge crest power densities (of the order of 10^19^ W/cm^2^) produced by the focused ultrashort pulses is sufficient to generate high-order nonlinearities in the medium, extracting electrons through a multiphoton absorption processes and spawning a hot plasma. This plasma is the source of thermal energy that leads the gold atoms to agglomerate in NP. Rather, large spherical clusters are formed by explosive diffusion of gold atoms.

Such a scheme is quite different from the one explaining the photoprecipitation of Au-NP in the same kind of samples under CW laser irradiation [30]. The CW irradiation conditions being more or less the same as previously described for Ag-NP and the Au-doped sample being the same as in the fs experiment, the result shown in Figure 5b is the local production of Au-NP at the surface of the sample, with a size distribution between 5 and 15 nm and a rather good space resolution of 20 μm. Although limited to the sample surface, this approach presents two main advantages over the IR fs experiment: firstly, CW lasers are obviously cheaper than a fs laser chain. Secondly, since one-photon absorption generates sufficient energy to extract electrons from the matrix, no carbonate additive is required here. In any case, both growing processes can be qualified as efficient to produce Au-NPs in a porous silica gel.

### Semiconductor nanoparticles in a xerogel

Semiconducting nanoparticles (SC-NP) are particularly suited to increase the linear refractive index of a glass, because their own refractive indices are among the highest [31]. For example, Bragg mirrors of high efficiency can be foreseen using a series of PbS-doped and nondoped regions in an optical fiber. Moreover, quantum confinement in SC-NP is the base for the well-known narrow and tunable light emission having a great potential in display and lighting technologies [32]. SC-NPs are also of particular interest for their high nonlinear refractive indices [11] and absorption coefficients [33], which depend on particle size too.

#### Cadmium sulfide nanoparticles

**Figure 6:**
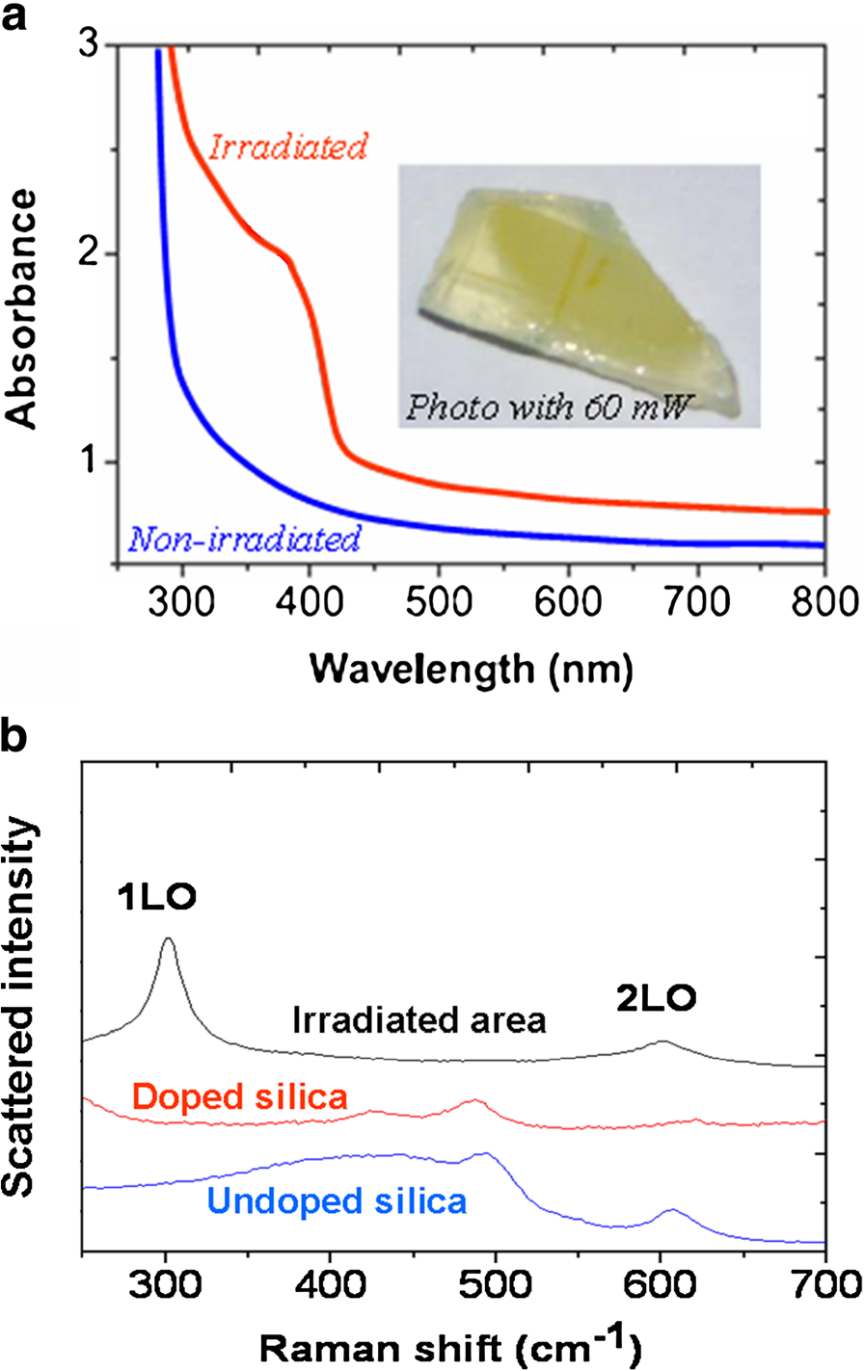
**Spectroscopic analysis of a xerogel impregnated with CdS precursors after fs irradiation.** (**a**) Absorption spectra in different zones with photograph of the sample irradiated with the highest laser power and (**b**) Raman spectra of different zones. (**a**) adapted from [37].

Xerogels of mean pore diameter of 5 nm were impregnated with a 0.56-M concentrated solution containing cadmium acetate and thiourea as precursors of CdS. After drying, they were irradiated under fs laser beam at 800 nm. Since neither the matrix nor the CdS precursors do absorb light linearly at this wavelength, the process involves essentially multiphoton absorption. The scanning setup enabled to cover a wide CdS-doped area in the bulk volume of the sample; with a mean power of 60 mW, a small part of the deposited energy (1,600 J/cm^2^) is absorbed and transformed into heat to break down precursor molecules and to form CdS-NP. This thermal process is however quite inefficient in the fs regime at low repetition rate [34]. Hence, a pale yellow color appeared when using the most concentrated doping solution and the highest laser power. The absorption spectrum of this area (Figure 6a) clearly shows the exciton band from 350 to 390 nm, which situates the NP size around 2 nm according to a tight-binding model [35]. Yellow traces, as well as the observation of an exciton peak in absorption spectra, are strong indices of the presence of CdS, but this presence and the nanoscale nature of the formed particles were formally attested by Raman spectroscopy. The quasi-resonant Raman spectrum of Figure 6b, taken by exciting the irradiated zone with a low-power laser beam at 473 nm, exhibits the well-known first longitudinal phonon bands of CdS (1LO) and its overtone (2LO). The ratio between 2LO and 1LO phonon band intensities allows estimating the CdS particle mean size [36], which is once again found close to 2 nm. It should be noted that this particle size remains more or less the same when the laser power is varied from 25 to 60 mW; only the NP concentration increases. Hence, this fs irradiation technique leads to produce, with a rather poor yield, only very small CdS particles, however localized in a microvolume of a width and depth defined by the laser waist (2 μm) and by the Rayleigh range (about 4 μm), respectively.

**Figure 7:**
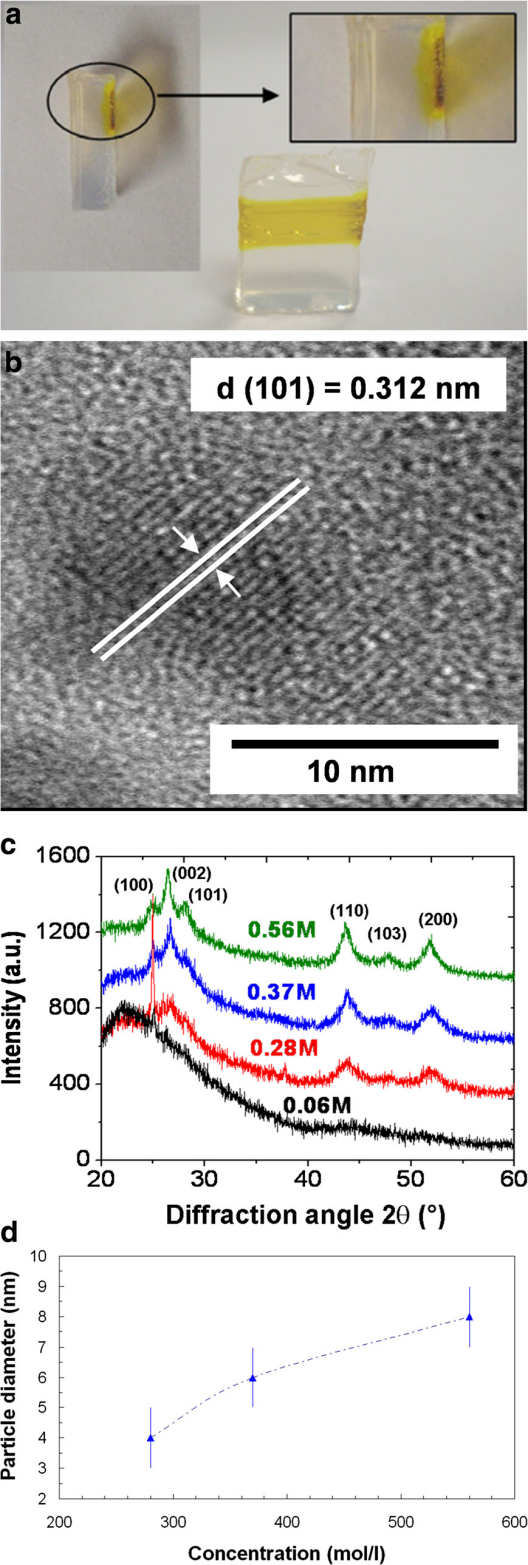
**Results obtained in a xerogel impregnated with CdS precursors after CW irradiation at 70 mW.** (**a**) Photographs of the irradiated surface of the sample. (**b**) HRTEM image of a single CdS NP. (**c**) XRD patterns obtained from the laser-irradiated zone for different doping concentrations and (**d**) the particle size evolution deduced from the width of the reflex (110). (**a**, **b**, and **c**) adapted from [37].

A better efficiency has been found in the local production of CdS NP through irradiation by a CW laser beam in the same kind of xerogels, impregnated with precursor solution of different concentrations [37]. In this case, the experimental setup yielded a deposited energy per surface area of 700 J/cm^2^, namely about half the one estimated in pulsed regime. However, in the CW regime, the wholeness of this energy could be transferred to the NP formation processes near the sample surface. From 200 J/cm^2^, a strong yellow coloration appeared under the surface inside the host matrix (Figure 7a). Although the large concentration of NP impedes the use of light absorption to characterize them precisely, structural techniques like TEM (Figure 7b) or X-ray diffraction (XRD, Figure 7c) could be used. Both of them show the hexagonal wurtzite structure of CdS, corresponding to large NPs and to a local temperature higher than 300°C during the laser irradiation [38,39]. The average particle diameter *D* could be evaluated using the width of (110) XRD reflex and the Debye-Scherrer formula:

(3) $$D=\frac{0.94\lambda }{B\cos\theta_{B}}$$

where *λ* is the X-ray wavelength, *B* is the full width at half maximum of the diffraction reflex (in radian), and *θ*_*B*_ its half-angle position. As shown in Figure 7d, this size is once again slightly higher than the mean pore size, which means that the efficient growing of CdS particles compels the matrix to a textural rearrangement.

#### Lead sulfide nanoparticles

**Figure 8:**
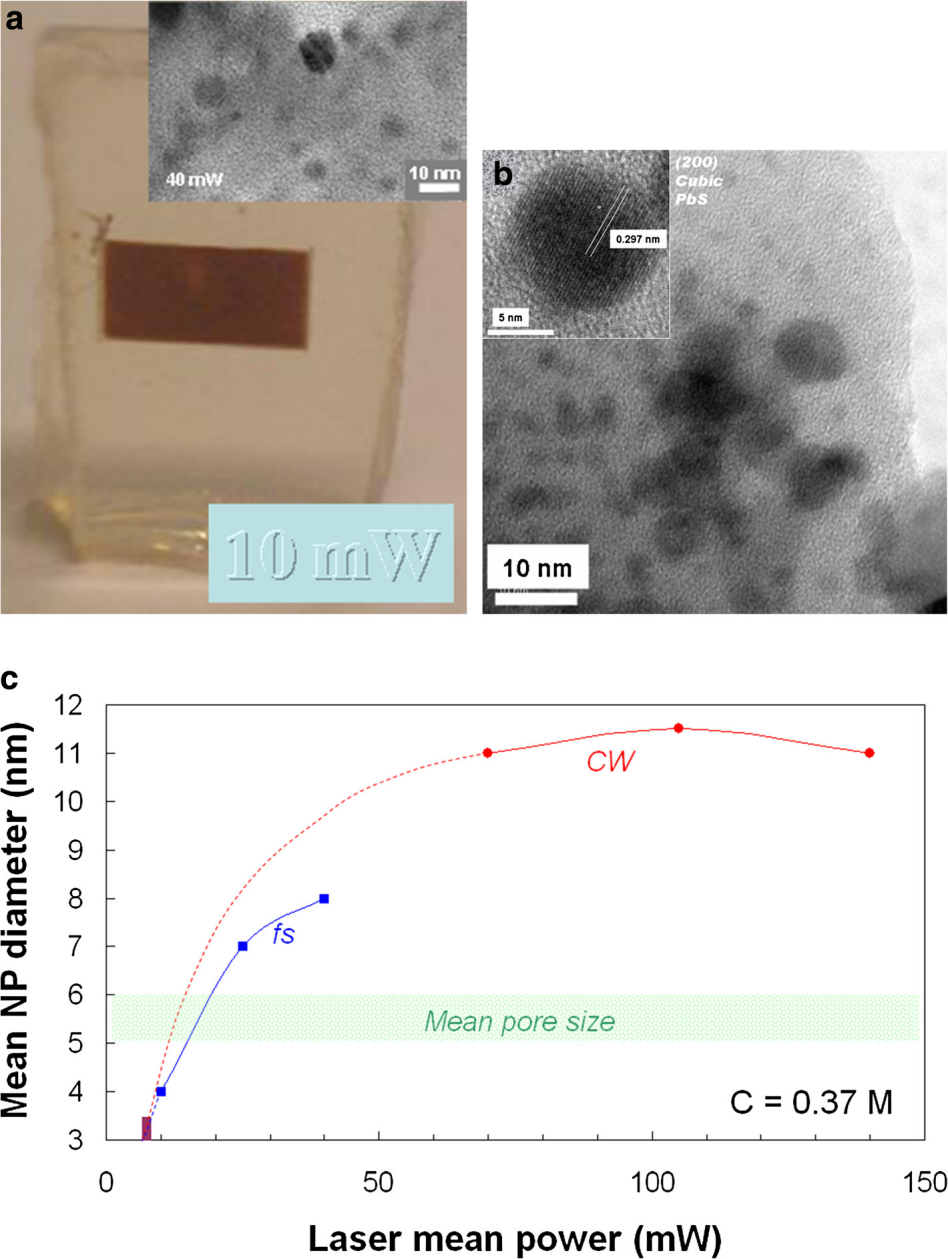
**Local growth of PbS NP in a xerogel impregnated with PbS precursors.** The doping solution had a concentration of 0.37 M in lead acetate. (**a**) Photograph of a sample fs irradiated at 10 mW and TEM image of NPs obtained after fs irradiation at 40 mW. (**b**) TEM and HRTEM images after CW irradiation at 140 mW. (**c**) Average particle size against the laser power in both regimes. The power threshold has been measured for the CW laser. Dotted lines are extrapolations. (**a** and **b**) adapted from [40] and [41], respectively.

Lead acetate and thiourea in aqueous solution have been used to impregnate a xerogel. Then, irradiation of this sample with fs pulses at 800 nm led to the rapid formation of PbS NP [40], which could be recognized not only by the brown coloration in Figure 8a but also by various characterization techniques (HRTEM, EDX analysis, electron diffraction, photoluminescence). Since the sample is initially transparent at 800 nm, the photogrowth process probably involves multiphoton absorption, but as soon as the first NP appear in the beam waist volume, one-photon absorption can occur and even becomes predominant. The TEM images (inset of Figure 8a) give particle sizes comprised between 5 and 12 nm for a given laser power of 40 mW, which is corroborated by XRD experiments. The evolution of the NP size with the laser power (Figure 8c, blue curve) shows that the crystal growth is not limited by the porosity, as it is always the case if the growth process is very efficient. The reason why photogrowth of PbS is found more efficient than in the case of CdS under fs irradiation at low repetition rate lies in the thermal origin of this process. In effect, the thermal energy liberated by one-photon absorption is fully sufficient for the precursor breakdown and for atom diffusion, whereas multiphoton absorption only acts as a starter.

An even darker and stronger coloration could be obtained by using a visible CW laser [41]. In this latter case, the high concentration of NP observed in the TEM image of Figure 8b is an indication of the process efficiency, as well as the particle size that overpasses the mean pore size. For the highest doping concentration (precursor solution 0.37 M), the mean NP diameter, estimated using PbS peaks in XRD pattern and Debye-Scherrer equation, seems to reach a maximum around 11 nm, namely about twice the pore size diameter. However, the particle size can be tuned down to 2 or 3 nm by decreasing the doping concentration.

**Figure 9:**
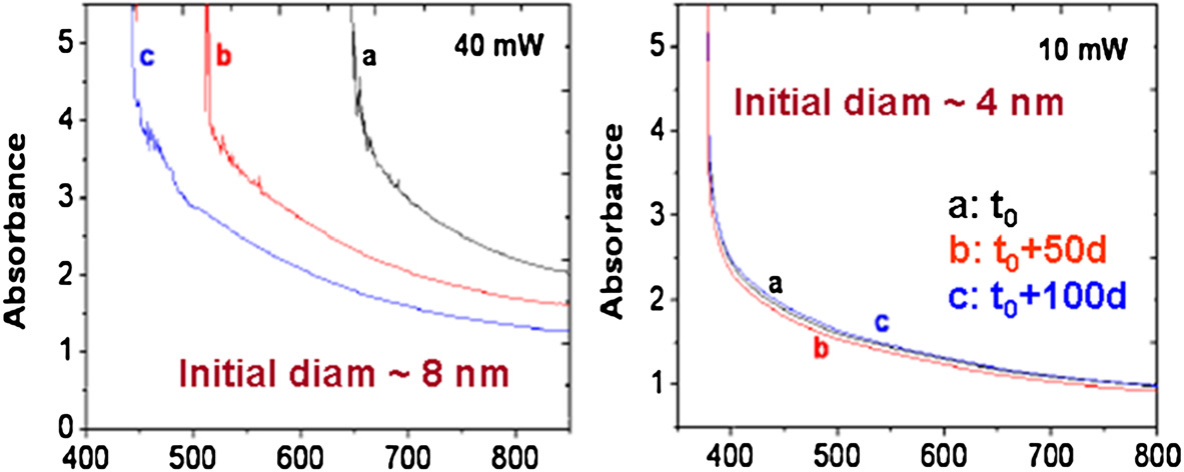
**Absorption spectra of PbS nanoparticles created by fs laser at different times after irradiation.** Left, sample irradiated with 40 mW, mean NP size 8 nm. Right, sample irradiated with 10 mW, mean NP size 4 nm. (Curve **a**) Just after irradiation, (curve **b**) 50 days after irradiation, and (curve **c**) 100 days after the initial irradiation. Adapted from [40].

One unfortunate feature of PbS NP is their affinity with oxygen to form PbO and PbSO_4_ compounds, leading to a poor stability of their optical properties [42]. It has been proposed [41] to adjust experimental conditions so as to produce NP without breaking silica pore walls, thus bringing the NP to fit the matrix pore size. In this case, PbS NPs are much longer protected by these walls from the atmosphere oxygen, and their optical properties remain unchanged for months (Figure 9).

## Conclusions

Our experience is rich of various photoinscriptions of NP in bulk xerogels. The growth of NPs depends on the laser power, the precursor's concentration, and a parameter which is difficult to control, the reaction or diffusion efficiency. If this parameter is high, the pore walls can be broken by the rapid expansion of the growing particles. Particle sizes obtained in different conditions are compiled in Table 1, where a correlation with the photoprocess efficiency is reported. With each type of laser having its own advantages, we now aim to provide an effective method to generate localized NP in a dense glass without post-annealing. In this remaining technological challenge lies the key for future photonic devices. However, densification of silica xerogels after the NP formation would require temperatures as high as 1,100°C, implying the NP destruction. So, the prospects should be turned toward the multicomponent glasses that have lower melting temperature and higher atom mobility. A possibility to avoid post-annealing treatment after fs irradiation would also be to use higher pulse cadency to provoke simultaneous metal ion reduction and heat accumulation [43]. It is expected that this work on xerogels will pave the way to future optical waveguiding devices.

**Table 1:** NP size: correlation with photoprocess efficiency

**Compound**	**Mean NP size (nm) CW**	**Mean NP size (nm) ns**	**Mean NP size (nm) fs**
Ag	10 to 20, ME		
CdS	4 to 8, HE	3 to 8^a^, LE	2 to 3, LE
Au	5 to 15, HE		20, HE
PbS	8 to 11, HE		4 to 8, HE

^a^According to [24]: pore size, 7 nm, precursors Cd nitrate + ammonium thiocyanate.

HE, high efficiency; ME, moderate efficiency; LE, low efficiency.

## Competing interests

The authors declare that they have no competing interests.

## Authors’ contributions

AC and HEH designed, analyzed, and performed most of the experiments. BC performed TEM experiments and wrote and corrected this report. MB is responsible for the correction of this report. AC, HEH, OC, BC, and MB have performed the interpretation and comparison of the results. All authors read and approved the final manuscript.
